# The ER Lumenal Hsp70 Protein FpLhs1 Is Important for Conidiation and Plant Infection in *Fusarium pseudograminearum*

**DOI:** 10.3389/fmicb.2019.01401

**Published:** 2019-06-28

**Authors:** Linlin Chen, Xuejing Geng, Yuming Ma, Jingya Zhao, Wenbo Chen, Xiaoping Xing, Yan Shi, Bingjian Sun, Honglian Li

**Affiliations:** ^1^College of Plant Protection, Henan Agricultural University, Zhengzhou, China; ^2^National Key Laboratory of Wheat and Maize Crop Science, Zhengzhou, China

**Keywords:** *Fusarium pseudograminearum*, Hsp70, FpLhs1, pathogenesis, protein secretion

## Abstract

Heat shock protein 70s (Hsp70s) are a class of molecular chaperones that are highly conserved and ubiquitous in organisms ranging from microorganisms to plants and humans. Hsp70s play key roles in cellular development and protecting living organisms from environmental stresses such as heat, drought, salinity, acidity, and cold. However, their functions in pathogenic fungi are largely unknown. Here, a total of 14 *FpHsp70* genes were identified in *Fusarium pseudograminearum*, including 3 in the mitochondria, 7 in the cytoplasm, 2 in the endoplasmic reticulum (ER), 1 in the nucleus, and 1 in the plastid. However, the exon–intron boundaries and protein motifs of the FpHsp70 have no consistency in the same subfamily. Expression analysis revealed that most *FpHsp70* genes were up-regulated during infection, implying that *FpHsp70* genes may play important roles in *F*. *pseudograminearum* pathogenicity. Furthermore, knockout of an ER lumenal Hsp70 homolog *FpLhs1* gene reduced growth, conidiation, and pathogenicity in *F*. *pseudograminearum*. These mutants also showed a defect in secretion of some proteins. Together, FpHsp70s might play essential roles in *F*. *pseudograminearum* and FpLhs1 is likely to act on the development and virulence by regulating protein secretion.

## Introduction

*Fusarium pseudograminearum* has been identified as a major causal agent of *Fusarium* crown rot (FCR), which is a chronic disease of wheat and barley in many cropping regions of the world (Mishra et al., 2006; Cepni et al., 2013; Aoki et al., 2015; Kazan and Gardiner, 2018). Particularly, FCR caused by *F*. *pseudograminearum* has become a serious issue in the Huanghuai wheat growing area of China (Li et al., 2012). This pathogen can also incite *Fusarium* head blight (FHB), especially if warm and humid conditions exist during anthesis. Symptom of the development of typical necrotic or bleached spikelets contaminated with the fungal toxin deoxynivalenol (DON) caused by *F*. *pseudograminearum* is similar to that caused by the principal FHB pathogen *Fusarium graminearum* (Obanor et al., 2013; Garmendia et al., 2018). However, little is known about the regulation of the virulence factors in *F*. *pseudograminearum*. Recently, the sequencing and comparative analyses of *F*. *pseudograminearum* genome have provided new insights into the processes involved in pathogen virulence (Gardiner et al., 2012, 2018).

Heat shock proteins, a family of highly conservative stress proteins, belong to a multi-gene family of proteins that differ in molecular size from 10 to 150 kDa and are found in all organisms from bacteria to humans. Members of the 70-kDa heat shock protein family (Hsp70) belong to a class of proteins termed molecular chaperones (Timperio et al., 2008; Richter et al., 2010; Tiwari et al., 2015; Ghazaei, 2017). Hsp70 proteins consist of two domains, a highly conserved 40-kDa N-terminal nucleotide-binding domain (NBD) and a less-conserved 25-kDa C-terminal substrate-binding domain (SBD), and a linker domain between NBD and SBD (Bertelsen et al., 2009; Sharma and Masison, 2009). Some eukaryotic Hsp70s have been reported to play important roles in various states of physiological and environmental stresses, such as infections, inflammation, cellular injury, or heat stress (Park and Seo, 2015; Tiwari et al., 2015). Furthermore, they localize in various cellular compartments, functioning in diverse cellular processes from protein folding to disassembly of protein complexes to protein translocation across membranes (Sharma and Masison, 2009; Duncan et al., 2015; Radons, 2016).

In the fungal system, Hsp70 proteins are highly conserved and play a major role in growth, morphogenesis, and various stress conditions. *Saccharomyces cerevisiae* contains two organelle-specific and six cytosolic Hsp70s, and the cytosolic Hsp70s are subdivided into two classes Ssa and Ssb. Each subfamily was considered to have the same functions. Ssa and Ssb play significant roles in posttranslational translocation (McClellan and Brodsky, 2000; Ast et al., 2013; Craig, 2018). Ssb interacts with most mitochondrial and endoplasmic reticulum (ER) proteins. Increased expression of Ssb will overcome the growth defect caused by inefficient mitochondrial protein translocation (Willmund et al., 2013; Wang and Chen, 2015). The ER lumenal Hsp70 protein Kar2p is essential for cellular homeostasis and participates in the transport of nascent polypeptides into the ER lumen, polypeptide folding, and the selection of misfolded proteins for degradation (Latterich and Schekman, 1994; Plemper et al., 1997). The other ER lumenal Hsp70 protein Lhs1p is not essential for viability, but *lhs1p* null mutant cells display a partial defect in posttranslational translocation and are also defective in the repair of misfolded proteins in the ER (Tyson and Stirling, 2000). Moreover, the chaperone activity of Kar2p is regulated by its intrinsic ATPase activity that can be stimulated by Lhs1p (Hale et al., 2010).

Many fundamental aspects of the translocation systems have been highly conserved in evolution. In *Magnaporthe oryzae*, MoSsb1 is important for the growth, conidiation, and full virulence of the blast fungus. It regulates the synthesis of nascent polypeptide chains through complex with other members of Hsp70s MoSsz1 and 40-kDa Hsp40 MoZuo1. Moreover, MoSsb1, MoSsz1, and MoZuo1 are all involved in the regulation of the CWI MAPK pathway by modulating MoMkk1 biosynthesis (Yang et al., 2018). The complex of Hsp70 proteins FgSsb, FgSsZ, and their cochaperone FgZuo regulates multiple stress responses and mycotoxin production *via* folding the soluble SNARE Vam7 and b2-tubulin in *F*. *graminearum* (Liu et al., 2017). In addition, both Lhs1 and Kar2 proteins localize in the ER and function in an unfolded protein response in *M*. *oryzae*. The *lhs1* mutants show a defect in the translocation of proteins across the ER membrane and effector protein secretion and reduce activities of extracellular enzymes, which lead to the pathogenicity reduction (Yi et al., 2009). Furthermore, in *Aspergillus terreus*, Hsp70 played roles for the antifungal amphotericin B (AmB) resistance (Blatzer et al., 2015). Although Hsp70 orthologs are conserved in eukaryotes, none of them have been characterized in *F*. *pseudograminearum*.

In this study, 14 *FpHsp70* genes were identified, and a comprehensive analysis was performed, including sequence characteristics, gene structures, and conserved motif analysis. The expression patterns of the *FpHsp70* genes indicated that most *FpHsp70* may play roles in the pathogenesis of *F*. *pseudograminearum*. Here, an ER lumenal Hsp70 protein FpLhs1 was further examined in *F*. *pseudograminearum*. The Δ*fplhs1* mutants exhibited defects in efficient growth, conidiation, conidial germination, and pathogenicity. We also found that FpLhs1 functioned in facilitating the secretion of proteins, including various extracellular enzymes. Taken together, our findings suggested that FpLhs1 might play a critical role in the development and virulence by acting on protein secretion in *F*. *pseudograminearum*.

## Materials and Methods

### Identification of *F*. *pseudograminearum Hsp70* Genes

The retrieved Hsp70 proteins from *M*. *oryzae* and *F*. *graminearum*^1^ were used as the query to search the *F*. *pseudograminearum* databases (whole genome, the predicted proteins, and genes) by BlastP and tBlastN algorithms (Altschul et al., 1990; Gardiner et al., 2018). Then, SMART and Pfam databases (Finn et al., 2016) were utilized to check the protein sequences of the candidate genes to confirm the presence of the Hsp70 domain and some other codomains. The exon–intron structures of the *FpHsp70* genes were displayed through Gene Structure Display Server 2.0^2^. The WoLF PSORT program^3^ was used to predict the subcellular localization of FpHsp70s. The MEME program (version 4.10.0^4^) was used to identify the conserved protein motifs of FpHsp70s.

### Expression Analysis of *FpHsp70* Genes

Expression data of *FpHsp70* genes were obtained from a transcriptome database. The process of transcriptome sequencing and assembly was described into: mycelia were harvested by conidia cultivation in potato dextrose liquid medium at 25°C in darkness for 12 h. A pot-culture experiment was used to harvest the infection samples. Millet was sterilized at 121°C for 20 min. Sterile millet was inoculated with *F*. *pseudograminearum* mycelia at 25°C for 7–10 days, and shook well every day until the mycelia overgrow in the millet. Then, 0.5% inoculation millet mixed with sterile soil for wheat growing. In controls, sterile millet was used. After 5 and 15 days at 25°C with 16-h light/8-h dark, the wheat roots from each pot were collected and washed thoroughly under running tap water and distilled water so that no soil particles remained. Two replications were performed. A total amount of 6 μg RNA per replication was used for the RNA sample preparations. The total transcriptome was sequenced by the Gene DeNovo Company (Guangzhou, China). Transcriptome data were processed by OmicShare Tools^5^. The raw data from the transcriptome analysis were submitted to the NCBI (Submission ID: SUB5545839), and the date will be released after May 31, 2020.

### Manipulation of *F*. *pseudograminearum*

The split-marker approach was used to generate gene-replacement constructs for the *FpLhs1* gene as described in our previous study (Wang et al., 2017). Primers were listed in Supplementary Table S1 and a schematic diagram of primers located for gene replacement with split-marker strategy and screening of mutant is shown in Supplementary Figure S1. To generate pKNTG-*FpLhs1* for gene complementary, the *FpLhs1* gene and a 1,387-bp upstream flanking genomic sequence of *FpLhs1* as the promoter of *FpLhs1* were amplified by PCR using primers cp-F and cp-R with termination codon missed in the 3′-terminal of *FpLhs1*. Then, the amplification was purified and digested by *Kpn*I and *Apa*I fused in *GFP* 3′-terminal of pKNTG. The ER marker was created by inserting a synthetic oligonucleotide encoding HDEL at the C-terminus of the *mCherry* genes and inserted into pDL2 vector. The polyethylene glycol (PEG)-mediated protoplast fungal transformation was performed as described previously (Liu and Friesen, 2012).

Genomic DNA was isolated from mycelia (Judelson et al., 1993) and screened for putative gene deletion mutants by PCR using the primers H852F/H850R, PF/H855R, H856F/PF, and NF/NR. The resequencing work was completed by the Gene DeNovo Company (Guangzhou, China). The sequence reads data from resequencing of WT and knockout strains were submitted to the NCBI (Submission ID: SUB5578218). The putative complementation was examined by PCR using primers NF and NR. A Nikon Ti-s Instrument was used to examine fluorescent conidia expressing GFP.

### Analysis of *F*. *pseudograminearum* Development

Mycelia and conidia of all strains were stored in 30% glycerin at −70°C. To evaluate growth, strains were subcultured twice and then grown on individual agar disks on PDA agar medium at 25°C. Mycelial morphology was observed 12 h later, and colony diameters were measured and photographed 3 days later; to assay mycelia growth upon different stress, different substances at indicated concentrations were added to solidified PDA medium, and then colony diameters were measured 3 days later. To explore conidia production, two agar disks from the edges of actively growing cultures were cultured in 100 ml of CMC medium at 150 rpm, 25°C in the dark for 7 days. Conidia were collected and counted. To explore conidia germination, 0.1 ml of 10^4^ conidia/ml suspension was prepared and cultured in sterile distilled water at 25°C in the dark for 6 and 10 h. All experiments were performed at least three times with over three replicates in each experiment. Data were analyzed using a *t*-test. A Nikon Ti-s Instrument was used to examine mycelia, conidia, and conidia germination.

### *F*. *pseudograminearum* Infection Assays

Mycelia of WT strain, mutants, and complementation were grown on PDA agar medium at 25°C for 3 days for plant infection assay. Five-centimeter-long wheat seedlings were collected and infected on coleoptile by fungal discs (5 mm in diameter). After 24 h, the fungal discs were removed, and lesion lengths of etiolated seedlings were photographed at 3 days post-inoculation (dpi). All experiments were performed at least three times with over five replicates in each experiment. Data were analyzed using a *t*-test. Malting barley seeds were planted in pots for 14 days, and leaves were cut off. A 5-mm-diameter fungal disc was infected on barely leaves, and the fungal disc was removed after 24 h. Lesion areas were photographed at 3 dpi. Infection assay by pot-culture experiment was conducted with 0.5% inoculation millet in sterile soil using pre-geminated wheat seeds. Wheat growth was photographed at 10 days. Hyphae infecting barley epidermal cells were viewed under a Nikon Ti-s Instrument for penetration assay.

### Secretory Protein Assays

Conidia of wild-type (WT) strain and mutants and complementation were collected and transferred to nitrogen-deficient liquid medium at 150 rpm, 25°C for 5 days. Fungal-culture medium was collected and dried by a vacuum freeze dryer. All secretory proteins were sequenced and analyzed by Applied Protein Technology (Shanghai, China). Protein digestion by trypsin was performed according to the filter-aided simple preparation (FASP) procedure described by Matthias Mann (Wisniewski et al., 2009). LC–MS/MS analysis was performed on a Q Exactive Mass Spectrometer (Thermo Scientific) that was coupled to Easy nLC (Proxeon Biosystems, now Thermo Fisher Scientific). The MS raw data for each sample were combined and searched using the MaxQuant 1.3.0.5 (Cox and Mann, 2008) software executing the Andromeda search engine against uniprot_Fusarium_pseudograminearum_13942_20180428.fasta database. The proteomics data were submitted to the iProX (integrated proteome resources) (subproject ID: IPX0001601001). PDX number is PXD013850. The gene ontology (GO) database was used to elucidate the functional classifications of biological process (BP), molecular function (MF), and cellular components (CCs)^6^ (Ashburner et al., 2000). To analyze the transcription levels of the selected secretory proteins, conidia were cultured in nitrogen-deficient liquid medium at 150 rpm, 25°C for 5 days. Total RNA of mycelia was extracted using the RNA simple Total RNA Kit (Tiangen, China) following the recommended protocol. The process of quantitative RT-PCR has been described in our previous study (Chen et al., 2014).

## Results

### Characterization of the Hsp70s in *F*. *pseudograminearum*

In previous studies, 13 and 7 putative Hsp70s have been identified in *M*. *oryzae* and *F*. *graminearum*, respectively (Yi et al., 2009; Liu et al., 2017). The sequences of the known proteins were used to conduct a BLAST search of *F*. *pseudograminearum* genome. A total of 16 putative *Hsp70* genes were initially obtained, out of which 2 putative sequences (without Hsp70 domain) were removed, based on the confirmation of Pfam and SMART scans. Detailed information about 14 *FpHsp70* genes is shown in Table 1. The transcripts of *FpHsp70* varied between 1,686 bp and 9,225 bp in length encoding proteins of 561–3074 amino acids, and the corresponding molecular weights were between 60.0 (FpHsp70-11) and 345.2 kDa (FpHsp70-13), and the isoelectric point (pI) values ranged from 4.94 (FpHsp70-10) to 8.44 (FpHsp70-5). More detailed information is shown in Table 1.

**TABLE 1:** Hsp70 family in *Fusarium pseudograminearum*.

**Gene name**	**Transcript name**	**Chr**	**Genome location**	**ORF length (bp)**	**Deduced protein**			
					**Length (AA)**	**Molecular weight (kDa)**	**PI**	**HSP70 domain**
*FpHsp70-1*	FPSE_00919	3	4525553	2031	676	73.1	5.61	48–650
*FpHsp70-2*	FPSE_02958	3	4083864	1728	575	63.9	7.56	111–426
*FpHsp70-3*	FPSE_04168	4	3393770	1977	658	75	4.99	110–422
*FpHsp70-4*	FPSE_04475	3	6691499	1779	592	67.2	6.27	96–422
*FpHsp70-5*	FPSE_06428	2	1811439	2238	745	81.6	8.44	287–591
*FpHsp70-6*	FPSE_07090	4	7835811	1686	561	63.3	6.12	158–421
*FpHsp70-7*	FPSE_09172	1	6316108	2337	778	86	4.98	3–722
*FpLhs1*	FPSE_10147	3	5767346	3012	1003	109.6	5.59	28–649
*FpHsp70-9*	FPSE_10232	2	7953131	1911	636	71.1	5.77	149–430
*FpKar2*	FPSE_10499	4	6486153	1992	663	72.5	4.94	42–646
*FpHsp70-11*	FPSE_11571	2	2145635	1686	561	60.0	4.97	17–456
*FpHsp70-12*	FPSE_11610	1	2617384	1962	653	71.2	4.99	4–611
*FpHsp70-13*	FPSE_11708	2	2268043	9225	3074	345.2	8.31	93–374
*FpHsp70-14*	FPSE_12049	1	2920399	1845	614	66.8	5.26	9–614

**FIGURE 1:**
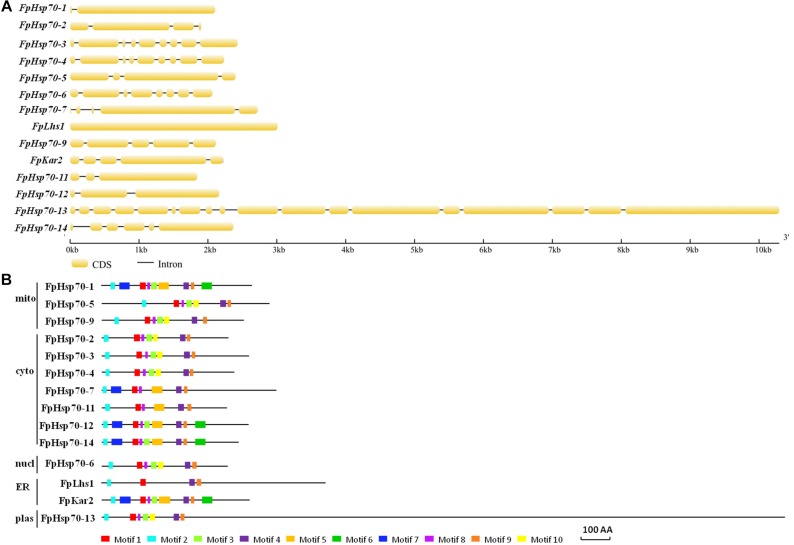
Gene structures and motif composition of FpHsp70s. **(A)** Exon–intron structure analyses of *FpHsp70* genes. The yellow sections represent exons, and the gray parts indicate introns. **(B)** Distribution of conserved motifs in FpHsp70. The boxes with different colors represent different motifs. mito, mitochondria; cyto, cytoplasm; nucl, nucleus; ER, endoplasmic reticulum; plas, plastid.

The FpHsp70 proteins were classified into five subfamilies, including three in the mitochondria (FpHsp70-1, -5, and -9), seven in the cytoplasm (FpHsp70-2, -3, -4, -7, -11, -12, and -14), two in the ER (FpLhs1 and FpKar2), one in the nucleus (FpHsp70-6), and one in the plastid (FpHsp70-13) (Figure 1B). In addition, the exon/intron organization in the coding sequence and the motifs of each FpHsp70 were identified. As shown in Figure 1, there were 1–4 introns in the mitochondria subfamily genes, 1–8 introns in the cytoplasm subfamily genes, and 0–17 introns in the ER, plastid, and nucleus subfamily genes (Figure 1A). Motifs in FpHsp70s were varied (Figure 1B). In brief, the exon–intron diagrams and protein motifs of the FpHsp70 were not consistent in *F*. *pseudograminearum*.

### Expression Patterns of *FpHsp70* Genes During Infection in *F*. *pseudograminearum*

**FIGURE 2:**
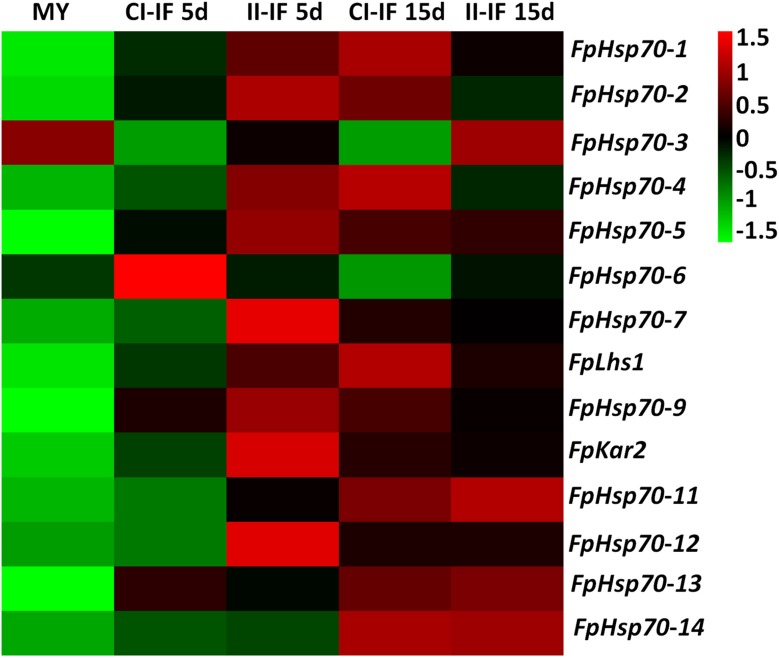
Expression profiles of *FpHsp70* genes. The color bar represents the relative expression values, ranging from green (–1.5) to red (1.5). MY, mycelia; CI-IF5 d and CI-IF15 d indicate samples from 5 to 15 days after infection of susceptible wheat *Guomai301*; II-IF5 d and II-IF15 d indicate samples from 5 to 15 days after infection of resistant wheat *Zhoumai24*.

The expression patterns of *FpHsp70* genes were determined during infection stages using the above-described transcriptional database (Figure 2 and Supplementary Table S2). *FpHsp70* genes were differentially expressed in the mycelia, but the expression levels of most *FpHsp70s* were up-regulated in the infection stages, except for *FpHsp70-3* and *FpHsp70-6*. Among these genes, *FpHsp70-12* had the highest expression levels at all stages, and *FpHsp70-4* was the most significantly up-regulated during infection, approximately 1,383 to 5,196 times up. In addition, expression of two ER *Hsp70* genes (*FpLhs1* and *FpKar2*) was up-regulated (two- to five- fold) by infection. In general, almost all *FpHsp70* genes were specifically expressed in infection, implying that these genes may play important roles in virulence of *F*. *pseudograminearum*.

### Knockout and Complementation of the *FpLhs1* Gene in *F*. *pseudograminearum*

**FIGURE 3:**
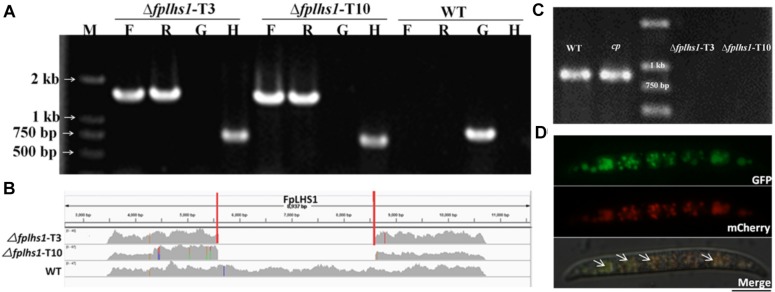
Construction of Δ*fplhs1* deletion mutants. **(A)** Verification of incorporation into genomic DNA by PCR using four pairs of primers, which was used to analyze *hygromycin* (H852F/H850R), upstream (PF/H855R), downstream (H856F/PF), and the *FpLhs1* gene (NF/NR) positivity. Amplified fragments were 750, 1467, 1521, and 885 bp. WT, wild-type strain WZ2-8; M, molecular markers; H, *hygromycin* gene; G, *FpLhs1* gene; F, upstream; R, downstream. **(B)** Resequencing analysis. *FpLhs1* is the only gene that was discarded in the two Δ*fplhs1* mutants. **(C)** Complementation of the Δ*fplhs1* deletion mutant. PCR assay using the primers NF and NR. Amplified fragment was 885 bp. WT, wild-type strain WZ2-8; M, molecular markers; *cp*, complemented strain. **(D)** The expression and localization of FpLhs1-GFP in Δ*fplhs1* mutants using its native promoter. mCherry-HDEL was used to visualize ER regions (middle column). Overlap of fluorescent signal indicates co-localization of the respective FpLhs1 in GFP fusion to mCherry-HDEL, indicated by the yellow fluorescent signal at the ER. Green line, GFP fluorescence intensity; red line, mCherry fluorescence intensity. Overlap of fluorescent signal was indicated by white arrows. Bars = 10 μm.

To explore the potential effects of these two proteins of FpLhs1 and FpKar2 in *F*. *pseudograminearum*, the target gene replacement construct was generated by the split marker approach and transformed into the WT strain *WZ2-8* using the PEG-mediated protoplast stable transformation method. In this study, two putative Δ*fplhs1* mutants, T3 and T10, were further confirmed by PCR and resequencing analysis. However, the *hygromycin* gene was lost in Δ*fplhs1* mutants after repeated subculture on PDA medium (Figures 3A,B). The FpLhs1–GFP fusion construct under the control of its native promoter was transformed for genetic complementation (*cp*). The putative complementation was examined by PCR and GFP fluorescence (Figure 3C). Microscopic observation showed that FpLhs1–GFP localized in the ER (Figure 3D). At the same time, over 50 transformants were screened for *Δfpkar2* mutant, but no knockout line was obtained.

### FpLhs1 Is Required for Normal Hyphal Growth of *F*. *pseudograminearum*

**FIGURE 4:**
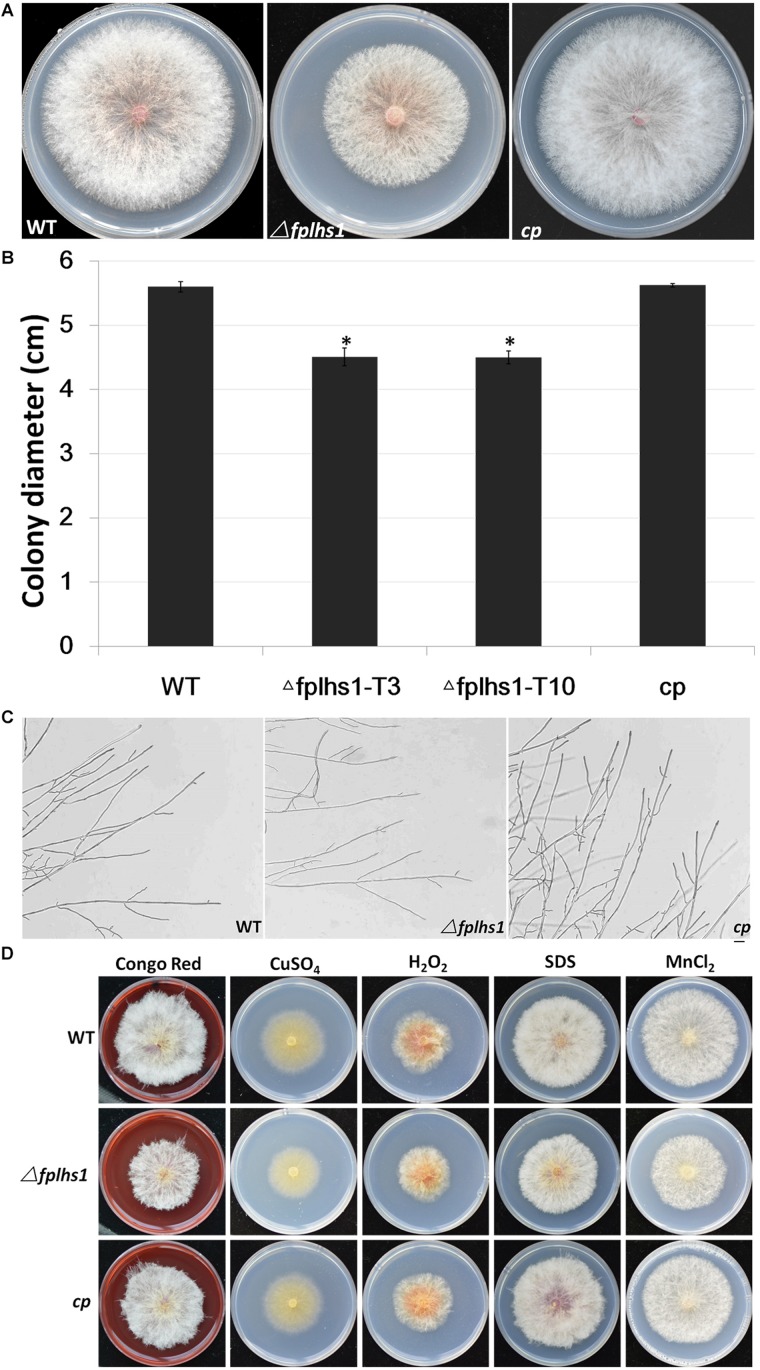
FpLhs1 contributes to the growth of *F*. *pseudograminearum*. **(A)** Colony of the WT, the Δ*fplhs1* mutants, and *cp* on PDA medium. Photographs were taken at 3 days after incubation (dai). **(B)** Colony diameters were measured at 3 dai. The data shown are representative of four colonies in each of three independent experiments. Standard errors were marked in brackets. ^*^P < 0.05 (*t-*test). **(C)** Hyphal tip growth and branching patterns of *F*. *pseudograminearum* grown on PDA medium for 12 h. Bars = 50 μm. **(D)** Colony of each strain on PDA with 25 mg/ml Congo Red, 1 mM CuSO_4_, 9 mM hydrogen peroxide, 10% SDS, or 10 mM MnCl_2_ for 3 days at 25°C.

To explore the role of FpLhs1 in vegetative growth, WT, the Δ*fplhs1* mutants, and the complemented transformant were cultured on PDA plates. After 3 days of incubation, the Δ*fplhs1* mutants exhibited a slightly reduced growth rate (Figures 4A,B). The Δ*fplhs1* mutants exhibited similar colony morphology with normal aerial hyphae and hyphal branches compared with the WT strain (Figure 4C). Furthermore, we tested the growth of Δ*fplhs1* mutants under different stress-inducing conditions. As shown in Figure 4D, on PDA supplemented with Congo Red, CuSO_4_, hydrogen peroxide, SDS, and MnCl_2_, the Δ*fplhs1* mutants showed similar tolerance to the WT strain. The results suggested that *FpLhs1* was responsible for hyphal growth, but not for the above stresses.

### Disruption of FpLhs1 Repressed Conidiation and Conidial Germination in *F*. *pseudograminearum*

**FIGURE 5:**
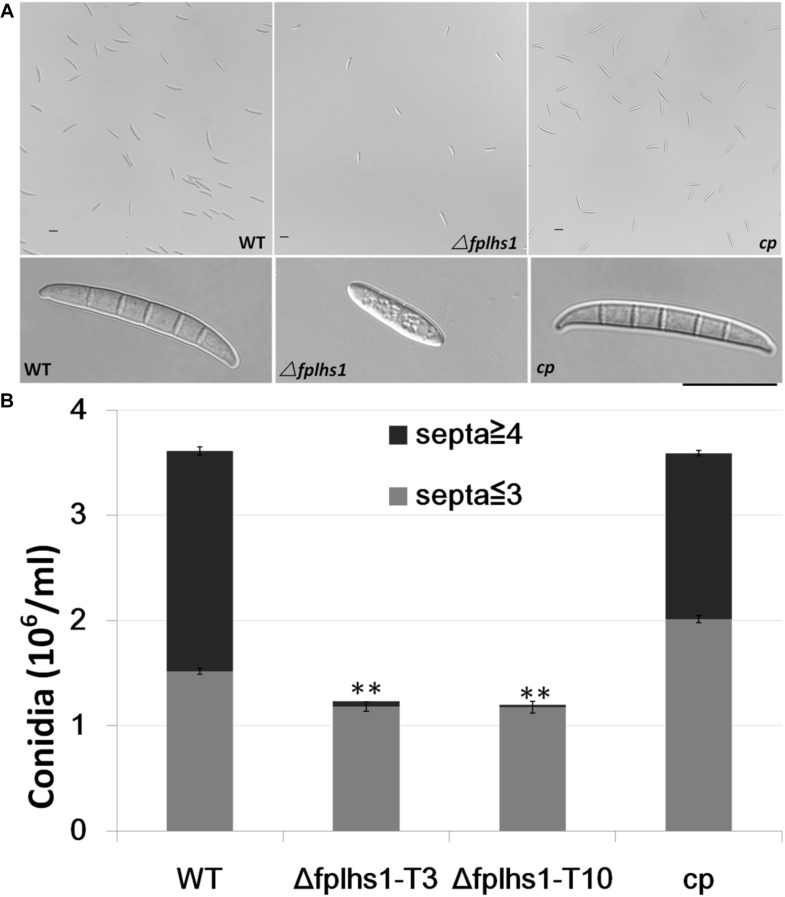
FpLhs1 contributes to the conidial production of *F*. *pseudograminearum*. **(A)** Conidial production and morphology of WT, Δ*fplhs1* mutants, and *cp* in 7-day-old carboxymethyl cellulose cultures. Bar = 20 μm. **(B)** Number of conidia produced by each line was measured at 7 dai. The data shown are representative of those of three separate experiments. The bars indicate the standard errors. ^∗∗^*P* < 0.01 (*t*-test).

To examine whether FpLhs1 plays roles in conidiation and conidial germination, WT and *Δfphls1* mutants were tested. Conidial production was assayed in CMC media. After 7 days of inoculation, the numbers of conidia in the *Δfphls1* mutants were reduced to approximately 35%, compared with that of the WT and the complemented strain (Figure 5). *FpLhs1* deletion also affected conidia morphology. The WT strain produced more than 50% conidia containing four and above septa. However, <5% of the conidia produced by the Δ*fplhs1* mutants contained four septa (Figure 5). The conidia length was also shorter in *Δfphls1* mutants (Figure 5). These results indicated that FpLhs1 was important for normal conidia production.

**FIGURE 6:**
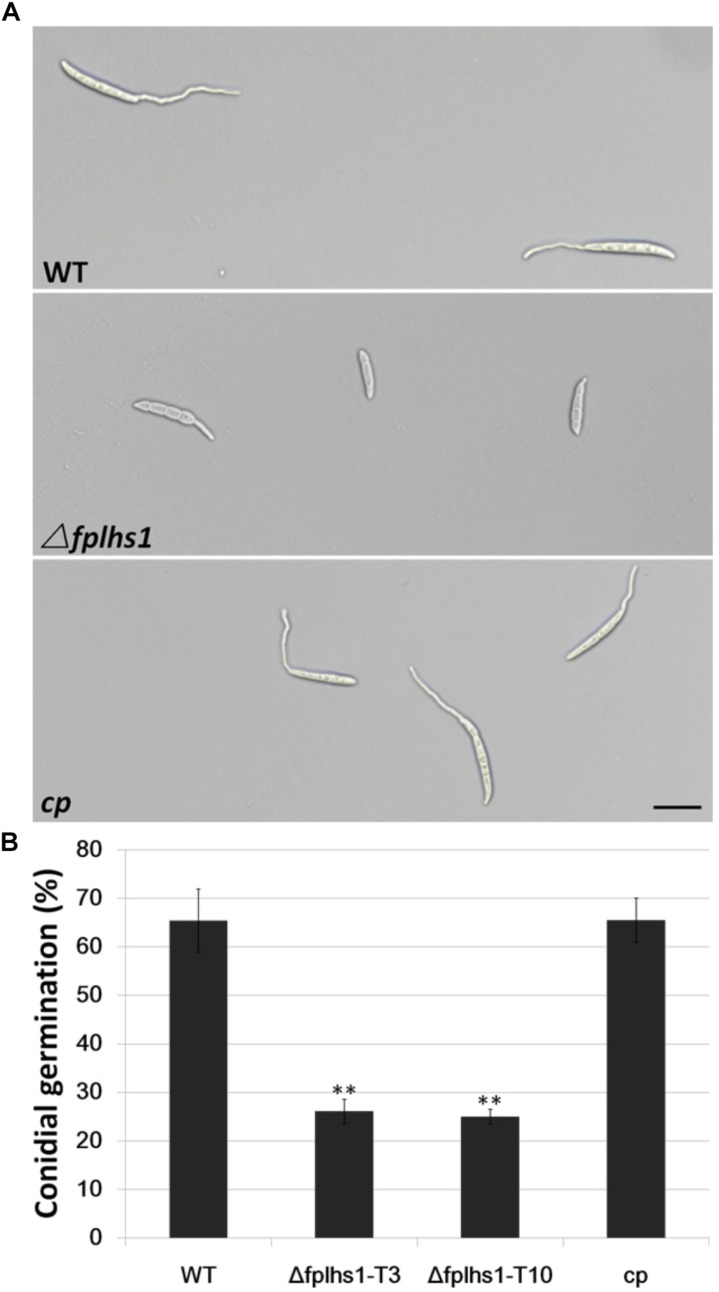
FpLhs1 contributes to the conidial germination of *F*. *pseudograminearum*. **(A)** Conidial germination in water of WT, Δ*fplhs1* mutants, and *cp* examined at 6 h after incubation (hai). Bar = 20 μm. **(B)** The germination rates of the conidia were measured at 6 hai in three independent biological replicates, each of which comprised at least five glass slides. The bars indicate the standard errors. ^∗∗^*P* < 0.01 (*t*-test).

To examine the role of FpLhs1 in conidial germination, conidia of WT, the *Δfphls1* mutants, and complementation strain were inoculated in sterile distilled water and observed for germination. It was found that over 65% conidia of the WT and complemented strain had visible germ tubes at 6 h, compared with 26.13 and 25% of that of the *Δfphls1* mutants T3 and T10, respectively (Figure 6). The results suggested that FpLhs1 played an important role in conidial germination.

### Disruption of FpLhs1 Reduced Pathogenicity in *F*. *pseudograminearum*

**FIGURE 7:**
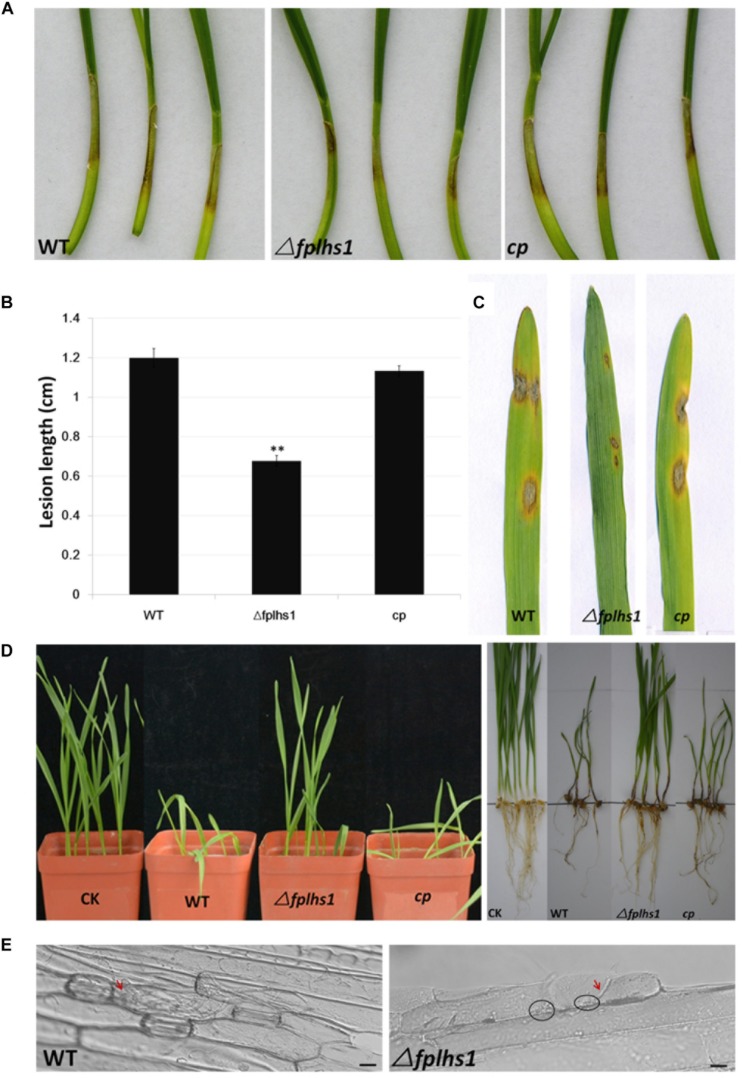
FpLhs1 contributes to the virulence of *F*. *pseudograminearum*. **(A)** Phenotypes of lesions on wheat hypocotyls inoculated with *F*. *pseudograminearum*. A susceptible wheat cultivar (*Aikang 58*) was inoculated with mycelia of each *F*. *pseudograminearum* line. Photographs representative of three independent experiments were taken at 3 days post infection (dpi). **(B)** Lengths of lesions on etiolated wheat hypocotyls were measured at 3 dpi in three independent biological replicates, each of which comprised at least five plants. The bars indicate the standard errors. ^∗∗^*P* < 0.01 (*t*-test). **(C)** Phenotypes of lesions on barley leaves inoculated with *F*. *pseudograminearum* mycelia of each line. Photographs were taken at 3 dpi. **(D)** Phenotypes of wheat growth and lesions on wheat roots inoculated with *F*. *pseudograminearum* millet inoculums. Photographs were taken at 10 dpi. **(E)** Representative micrographs of barley leaves that were inoculated with *F*. *pseudograminearum* mycelia of WT and Δ*fplhs1* mutant at 24 h post-infection. Infectious mycelia are indicated by red arrows, and deposits are indicated by black circles. Bars = 10 μm.

To clarify whether FpLhs1 was involved in the virulence of *F*. *pseudograminearum*, both wheat coleoptiles and leaves were selected to test the pathogenicity. Wheat coleoptiles were point inoculated with mycelia disks, and the lesion size upon infection with Δ*fplhs1* mutants was reduced by 56.5% (comparing to WT) (Figures 7A,B). Next, we performed a leaf-inoculation assay to validate the result. The Δ*fplhs1* mutants were less virulent than the WT and cp (Figure 7C). Disease symptoms were further observed in a pot inoculation experiment with mycelia prepared from WT, Δ*fplhs1* mutants, and *cp*. Most wheat seedlings were infected and slow-growing at 10 dpi after inoculation with the WT and complement; in contrast, wheat seedlings showed mild symptom after inoculation with the Δ*fplhs1* mutants (Figure 7D). Thus, deletion of *FpLhs1* reduced virulence of *F*. *pseudograminearum*.

To exclude the possibility that the observed reduction in virulence was a consequence of a reduction in growth rate (Figure 4A), we detected hyphae infecting barley leaf epidermal cells (Figure 7E). Many infected hyphae were observed upon infection with WT. In contrast, little Δ*fplhs1* mutant hyphae were observed in the infected leaf epidemical cells. Rather, deposits were evident at the interface between the pathogen and the barley, indicating that hyphal penetration might be impaired (Figure 7E). These results confirmed that FpLhs1 was important for pathogenicity in *F*. *pseudograminearum*.

### Involvement of FpLhs1 in the Regulation of *F*. *pseudograminearum* Protein Secretion

**FIGURE 8:**
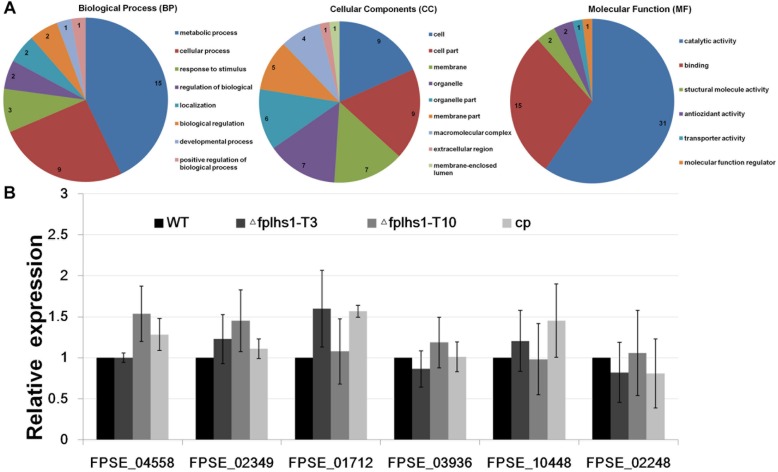
**(A)** GO categories analysis of these low-content proteins in two Δ*fplhs1* mutants. **(B)** qRT-PCR measurement of the relative transcript levels in control (WT and *cp*) and two Δ*fplhs1* mutants. The relative expression levels are calculated using *TEF1* as the reference gene. The bars indicate standard errors and stars above bars indicate the significant difference from the WT value.

We separately identified the secretory proteome of the WT strain and two Δ*fplhs1* mutants. Compared with the WT, a total of 60 proteins showed significantly reduced quantity in two Δ*fplhs1* mutants (Figure 8A, Supplementary Figure S2, and Supplementary Table S3). GO terms were applied to classify proteins into BP, MF, and CC according to their functional annotation. In the BP category, most of the proteins were in two major subcategories, namely, metabolic process and cellular process. In the MF category, proteins mapping to GO terms for catalytic activity and binding protein binding were the most abundant. For the CC ontology, proteins were dispersed in different Go terms.

To exclude the possibility that *FpLhs1* deletion interfered with the transcription of these secretion protein genes, the expression levels of the six selected secretion protein genes in WT and two Δ*fplhs1* mutants were assayed by qRT-PCR. As depicted in Figure 8B, the transcriptional level of all these genes had no obvious difference between in the WT strain and two Δ*fplhs1* mutants. The above results suggested that FpLhs1 might affect the protein secretion in *F*. *pseudograminearum*.

## Discussion

Heat shock protein 70s are ubiquitous molecular chaperones that play vital roles during eukaryote growth and development and protect the cellular machinery under stress conditions (Yu et al., 2015). The biological functions of Hsp70s have been studied in many fungi, such as *M*. *oryzae*, *F*. *graminearum*, and *S*. *cerevisiae* (Yam et al., 2005; Yi et al., 2009; Liu et al., 2017). However, no effort has been made to elucidate Hsp70s in *F*. *pseudograminearum*. In this study, a comprehensive genome-wide analysis of the *FpHsp70* gene family and the further function of FpLhs1 in *F*. *pseudograminearum* were carried out, and the results would increase our understanding of the regulatory mechanisms of the *F*. *pseudograminearum* pathogenesis.

In eukaryotes, Hsp70s play roles in diverse cellular processes from protein folding to protein translocation when they present in different cellular compartments (Clerico et al., 2015). In *S*. *cerevisiae*, the Ssa or Ssb subfamily share similar sequences and functions, but not any one of the Ssbs can compensate for essential Ssa function, which exemplifies the overlapping and distinct functions of the same cytosolic Hsp70 (Sharma and Masison, 2009). In the current study, a total of 14 *FpHsp70* genes were identified in *F*. *pseudograminearum*. Compared to *M*. *oryzae* and *F*. *graminearum*, all members of the Hsp70 gene family were found in *F*. *pseudograminearum*. FpHsp70 proteins were classified into five subfamilies by their subcellular localization, including three in the mitochondria, seven in the cytoplasm, two in the ER, one in the nucleus, and one in the plastid. However, the exon–intron boundaries and protein motifs of the FpHsp70 had no consistency in *F*. *pseudograminearum*. In plants, the intron pattern and protein motifs of Hsp70s are related to the gene function, since the Hsp70s in the same subfamily contain similar intron pattern and protein motifs but differ significantly among the different subfamilies (Chen et al., 2018).

Heat shock protein 70s chaperones, with their co-chaperones, comprise a set of abundant cellular machines that assist a large variety of protein folding processes in almost all cellular rearrangements (Mayer and Bukau, 2005). Although little is known about functions of Hsp70 chaperones in filamentous fungi, some studies have shown their function in different physiological processes. For example, Hsp70 orthologous associated with extracellular pH changes or AmB resistance in *A*. *terreus* and regulated multiple stress responses and mycotoxin production in *F*. *graminearum* (Blatzer et al., 2015; Liu et al., 2017). In the present study, we found that most *FpHsp70* genes were up-regulated in the infection, implying that FpHsp70 may play important roles in virulence of *F*. *pseudograminearum*.

In *S*. *cerevisiae*, evidence suggests that two ER Hsp70s, Kar2p and Lhs1p, functionally overlap in protein translocation into the ER and protein folding in the ER. Kar2p interacts with Sec63p and the nucleotide exchange factor Sil1p. Unlike Kar2p, Lhs1p had no measurable ATPase activity and was unaffected by the presence of either the Sec63p, Sil1p, or both in combination. However, Sil1p and Lhs1p act as equivalent nucleotide exchange factors for Kar2p (Tyson and Stirling, 2000; Steel et al., 2004; Hale et al., 2010). Kar2 and Lhs1 are conserved throughout eukaryotes. Here, we showed that the FpLhs1 was necessary for proper growth, conidiation, and pathogenicity of *F*. *pseudograminearum*. We also analyzed the secretory proteomes of Δ*fplhs1* mutants, and lots of proteins showed significantly reduced quantity. This further added to the previous findings that Lhs1 regulated the translocation of proteins across the ER membrane and reduced activities of extracellular enzymes in *M*. *oryzae* (Yi et al., 2009). However, compared with the WT strain, few effector homologs showed significant reduction in the secretion of Δ*fplhs1* mutants. The information was not enough to verify that effector secretion was regulated by FpLhs1. Since the expression dynamics of most effectors depend on host compatibility, we are not sure if effectors were expressed in the nitrogen-deficient liquid medium (Dou and Zhou, 2012). In conclusion, the FpLhs1 is localized to the ER and may be involved in protein secretion. The protein positively influences conidiation and pathogenesis in *F*. *pseudograminearum*.

In addition to these experiments, we attempted to delete the other ER lumenal Hsp70 protein FpKar2 in *F*. *pseudograminearum*, but no stable genetic transformant was obtained. However, more studies of the regulation mechanisms of different FpHsp70 will be needed to further explain functions of Hsp70 members in the *F*. *pseudograminearum*.

## Author Contributions

HL, WC, and LC conceived the study, participated in its design, and coordinated and drafted the manuscript. BS and XX performed the bioinformatics analysis. XG, YM, and JZ performed the experiments. YS performed the proteomics. All of the authors participated in the data analysis and interpretation, and read and approved the final manuscript.

## Conflict of Interest Statement

The authors declare that the research was conducted in the absence of any commercial or financial relationships that could be construed as a potential conflict of interest.

## Footnotes

1 http://www.ncbi.nlm.nih.gov

2 http://gsds.cbi.pku.edu.cn

3 https://wolfpsort.hgc.jp/

4 http://meme-suite.org/

5 www.omicshare.com/tools

6 http://www.geneontology.org
