# Supplementary material for: The ER Lumenal Hsp70 Protein FpLhs1 Is Important for Conidiation and Plant Infection in Fusarium pseudograminearum
Source: Front Microbiol. 2019 Jun 28;10:1401. doi: 10.3389/fmicb.2019.01401 (PMC6611370; doi:10.3389/fmicb.2019.01401)
Supplement: Supplementary file 1 [file Data_Sheet_1.pdf]

Supplementary Material

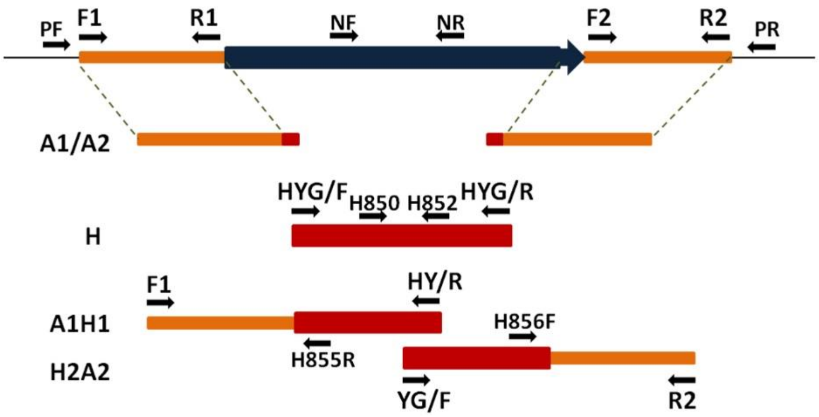

Supporting Information FIGURE S1. Schematic diagram of genome region of FpLhs1 and primers located for gene replacement with split-marker strategy and screening of mutant.

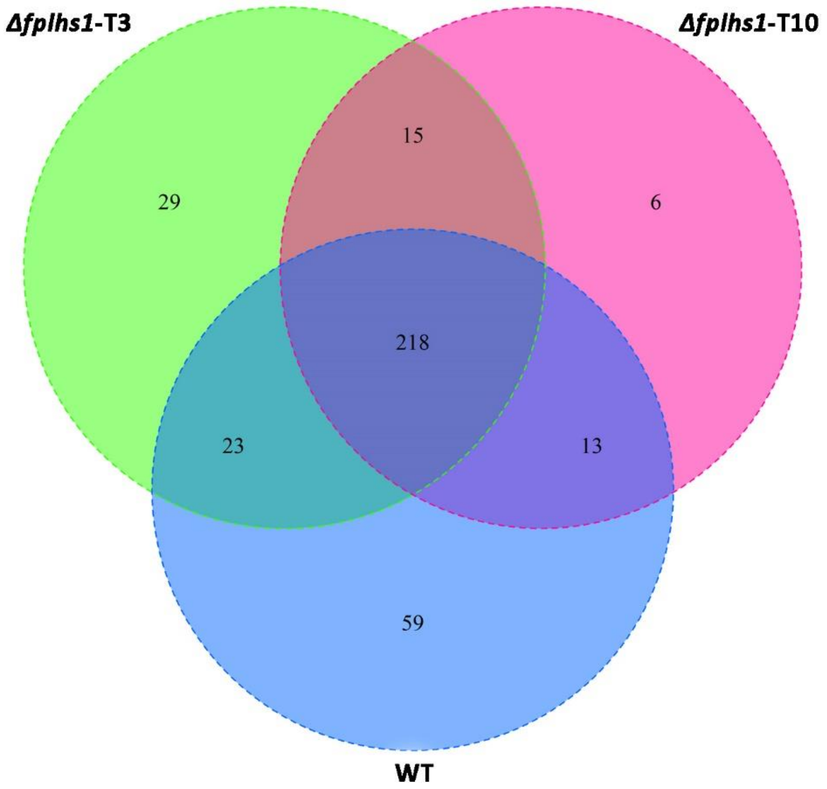

Supporting Information FIGURE S2. Venn diagram depicting overlap proteins between wild type and *Δfplhs1* mutants.
