# Supplementary material for: The ER Lumenal Hsp70 Protein FpLhs1 Is Important for Conidiation and Plant Infection in Fusarium pseudograminearum
Source: Front Microbiol. 2019 Jun 28;10:1401. doi: 10.3389/fmicb.2019.01401 (PMC6611370; doi:10.3389/fmicb.2019.01401)
Supplement: Supplementary file 2 [file Table_1.DOCX]

**Supporting Information Table S1.** Primers used in the study

| Primer | Sequence (5΄→3΄) |
| --- | --- |
| F1 | GTGCAGTGATGTGTCTATCACATG |
| R1 | CAATATCATCTTCTGTCGACGATGGTGGTTTGGTGGGGGTG |
| F2 | ATAGAGTAGATGCCGACCGCGGGTTCACGATGACGTGTTGACTTTTG |
| R2 | TCTGTACCATCATCGAGGAGTGAG |
| NF | TCCGCTCCATTGTTCTTAC |
| NR | TGGTCTCCCACTTCACGCC |
| PF | ATCTCCCGAGGGCAACTG |
| PR | ATCTCCTTGAATCTCCCGAG |
| cp-F | aactGGTACCGTACTCCCAAACATGGCATG |
| cp-R | aattGGGCCCTAGTTCGTCGTGAGTCTGCT |
| HYG/F | GGCTTGGCTGGAGCTAGTGGAGGTCAA |
| HYG/R | GTATTGACCGATTCCTTGCGGTCCGAA |
| HY/R | GTATTGACCGATTCCTTGCGGTCCGAA |
| YG/F | GATGTAGGAGGGCGTGGATATGTCCT |
| H855R | GCTGATCTGACCAGTTGC |
| H856F | GTCGATGCGACGCAATCGT |
| H852F | TTCCTCCCTTTATTTCAGATTCAA |
| H850R | ATGTTGGCGACCTCGTATTGG |
