# Supplementary material for: The ER Lumenal Hsp70 Protein FpLhs1 Is Important for Conidiation and Plant Infection in Fusarium pseudograminearum
Source: Front Microbiol. 2019 Jun 28;10:1401. doi: 10.3389/fmicb.2019.01401 (PMC6611370; doi:10.3389/fmicb.2019.01401)
Supplement: Supplementary file 3 [file Table_2.DOCX]

| Gene Name | MY (replication 1) | MY (replication 2) | CI-IF 5d (replication 1) | CI-IF 5d (replication 2) | II-IF 5d (replication 1) | II-IF 5d (replication 2) | CI-IF 15d (replication 1) | CI-IF 15d (replication 2) | II-IF 15d (replication 1) | II-IF 15d (replication 2) |
| --- | --- | --- | --- | --- | --- | --- | --- | --- | --- | --- |
| *FpHSP70*-*1* | 294.41 | 212.28 | 260.34 | 376.8 | 455.37 | 328.07 | 483.83 | 249.41 | 311.87 | 365.82 |
| *FpHSP70*-*2* | 5.12 | 10.88 | 192.33 | 114.82 | 139.21 | 369.96 | 412.16 | 184.56 | 186.99 | 100.04 |
| *FpHSP70*-*3* | 1 | 1.3 | 0 | 0 | 0 | 0 | 0 | 1.31 | 1.22 | 1.24 |
| *FpHSP70*-*4* | 0 | 0.07 | 75.05 | 21.79 | 31.86 | 331.9 | 199.1 | 112.06 | 102.05 | 41.56 |
| *FpHSP70*-*5* | 133.35 | 227.26 | 361.8 | 287.03 | 334.27 | 410.92 | 485.84 | 353.92 | 421.68 | 300.53 |
| *FpHSP70*-*6* | 0.94 | 0.89 | 7.62 | 0 | 0 | 0 | 0.74 | 1.57 | 1.47 | 1 |
| *FpHSP70*-*7* | 55.5 | 69.27 | 169.63 | 131.58 | 437.37 | 149.03 | 852.94 | 165.33 | 289.77 | 226.47 |
| *FpLhs1* | 39.07 | 35.63 | 61 | 99.17 | 105.44 | 168.46 | 124.29 | 98 | 98.56 | 101.59 |
| *FpHSP70*-*9* | 32.51 | 26.06 | 19.72 | 60.11 | 35.82 | 46.95 | 60.38 | 28.51 | 41.84 | 36.5 |
| *FpKar2* | 516.78 | 317.12 | 905.18 | 1105.69 | 1378.29 | 1565.75 | 2496.47 | 1971.32 | 1374.86 | 1341.25 |
| *FpHSP70*-*11* | 38.39 | 49.75 | 57.12 | 67.56 | 245.16 | 27.21 | 103.89 | 100.47 | 127.88 | 179.62 |
| *FpHSP70*-*12* | 1338.41 | 1878.82 | 1590.61 | 2107.53 | 4179.45 | 1593.55 | 6976.46 | 1504.04 | 3152.7 | 2590.45 |
| *FpHSP70*-*13* | 9.35 | 6.76 | 15.07 | 22.68 | 32.94 | 8.59 | 21.51 | 12.32 | 25.51 | 17.65 |
| *FpHSP70*-*14* | 790.05 | 600.57 | 924.52 | 937.56 | 2178.09 | 1100.67 | 1196.75 | 747.75 | 1713.54 | 1522.59 |

**Supporting Information Table S2.** Transcriptome data (FPKM values) of *FpHSP70* genes
